# Parent-child attachment mediates the association between parental conflict perceptions and suicide intention: a cross-sectional survey among middle school students in China

**DOI:** 10.3389/fpubh.2024.1332095

**Published:** 2024-04-03

**Authors:** Jiana Wang, Kun Chen, Xinyuan Huang, Zhiyu Jin, Jing He, Bingsong Han, Lin Feng, Nana Meng, Cong Yang, Pin Yao, Zhe Li

**Affiliations:** ^1^School of Public Health, Health Science Center, Ningbo University, Ningbo, Zhejiang, China; ^2^Department of Social Medicine, School of Public Health, China Medical University, Shenyang, Liaoning, China; ^3^Department of Health Management, Department of Ultrasound, Shengjing Hospital of China Medical University, Shenyang, Liaoning, China; ^4^Department of Anesthesiology, The Fourth Affiliated Hospital of China Medical University, Shenyang, Liaoning, China

**Keywords:** suicide intention, parent-child attachment, parental perception conflicts, adolescents, mediation analysis

## Abstract

**Introduction:**

Adolescent suicide is a prevalent issue globally, with various factors contributing to this phenomenon. This study aimed to investigate these factors and their interrelationships to better understand the causes of adolescent suicide and provide evidence for its prevention.

**Methods:**

This study conducted among middle school students in Liaoning Province, China, from April to May 2016, A cross-sectional survey was administered to 1,028 students aged 10–19, using instruments such as the Behavior Questionnaire-Revised (SBQ-R), Children's Perception of Interparental Conflict Scale (CPIC), and revised version of Inventory of Parent Attachment (IPPA-R).

**Result:**

Binary logistic regression analysis revealed that adolescents aged 15–19, adolescents with strong perceptions of parental conflict were at high risk of suicide intention. Adolescents living in rural areas, adolescents with high mother-child attachment, adolescents with high father-child attachment were at low risk of suicide intention. Furthermore, parent-child attachment played a mediating role between two dimensions of parental conflict perception (resolved situations and response effect) and suicide intention.

**Discussion:**

The study concludes that adolescents living in urban areas, older adolescents, adolescents with a high level of parental conflict intensity, and those with low levels of parent-child attachment are at high risk of suicide intention. parent-child attachment played a mediating role between two dimensions of parental conflict perception (resolved situations and response effect) and suicide intention. Interventions aimed at reducing family conflicts and improving parent-child relationships are recommended to decrease the incidence of adolescent suicide.

## Introduction

Suicide is a major public health problem that has become more serious worldwide in recent years, and suicidal behavior develops gradually from suicidal ideation, suicide attempts (1). Suicidal ideation is defined as an individual's idea of harming himself or herself or committing suicide and is a risk factor for suicide attempts and suicide completion (2). And it is a strong risk factor for suicide death (3). According to relevant studies, the occurrence of suicidal behavior and the generation of suicidal intentions run through all ages of a person, but they are especially common in adolescents (4). In the context of our research site, adolescents face a range of issues. Adolescents are in the important period of rapidly physical development. Thus, they tend to be energetic and have a good ability of acceptance of new things. However, they are likely to have psychological sensitivity to undesirable information, leading various adverse emotions, such as anxiety, depression, and problematic behaviors, including violence, bullying, and self-injury or even suicidal behaviors. Their physical and psychological welfare are influenced by various factors, including family conflicts, academic pressures, social issues, etc. In urban areas, adolescents may face greater academic and social pressures, while in rural areas, they may face more economic pressures and lack of resources. These issues can impact their mental health, thereby increasing their risk of suicide. According to a study by the World Health Organization, one person dies by suicide every 40 s in the world, and it is estimated that more than 800,000 people die by suicide every year (5). Suicide is the second leading cause of death among young people aged 15–29 years worldwide (6). According to a relevant study, suicide was the second cause of death next to traffic accidents among urban adolescents, and ranked third after traffic accidents and drowning among rural adolescents (5). Suicide seriously endangers the lives and health of young people and it causes serious losses to individuals, families, and even society (7). Therefore, the problem of suicide among Chinese adolescents should be paid close attention.

Parental conflict is defined as the frequency and intensity of conflicting behavior between parents, such as arguments, disagreements, quarrels, including physical and verbal attacks (8). Perceived parental conflict, which reflects the child's own cognitive and emotional processes toward parental conflict, has received frequent attention (9). Relevant studies have shown that parental conflict is closely related to various problem behaviors of adolescents, including smoking, alcoholism and suicide (10, 11). One study showed that hostile parental conflict has a negative impact on the development of behavioral problems in adolescents (12). Adolescents are in a critical period of physical and psychological development, facing various growing pains and pressures, the impact of parental conflict on adolescents' physical and mental health and problematic behaviors is obvious and worthy of attention. Exploring about perceived parental conflict on adolescent suicidal intention is of great significance.

According to the Attachment Theory, the quality of early relationships, particularly those with parents, can significantly influence an individual's psychological development and emotional wellbeing. The concept of attachment was first proposed by Bowby, who defined attachment as the tendency of individuals to form strong emotional bonds with specific other people, which enables individuals to maintain inseparable relationships with their caregivers early in life, thus obtaining the conditions for survival and development (13). Parent-child Attachment is defined as an attachment between children and their parents. Parent-child attachment is strongly linked to adolescent suicidal intentions. There is a study shown that the insecurity of parent-child attachment can be an important factor in adolescent suicidal behavior (14). A similar conclusion was reached in a study by Audino A: The probability of suicidal behavior is related to the quality of parent-child attachment, and the higher the quality of parent-child attachment, the lower the probability of suicidal behavior (15). A strong parent-child attachment has been shown to promote resilience and act as a protective factor against various negative outcomes, including suicidal ideation (16). In addition, during the special period of adolescence, adolescents are prone to a variety of problematic behaviors and psychological disorders, such as declining academic performance (17), initiation of drug use (18), or self-concept problems (19). However, parental concern or indifference at this time may be a protective or risk factor for adolescent problems (18, 20). Some studies have shown that parental responses to adolescents (affection, involvement, and trust) appear to have positive implications for the development of adolescent psychological autonomy and the prevention of deviant behavior (21, 22). A study in the United States showed that parents who are responsive to their children's needs allow their children to bond with them and feel loved by their family (23–25). Similar results were obtained in Europe (26, 27). Even more studies have further shown that relationships between parents and adolescent children based on positive responses protect them from problems (18, 28). Moreover, for adolescents, some studies have shown that, high-quality parent-child attachment will contribute to high social adaptability and cultivate positive psychological qualities, in turn, it reduces various negative emotions and bad behaviors including suicide (29). On the contrary, low-quality parent-child attachment will be one of the important factors that produce various negative emotions such as anxiety, depression. And, studies have shown that those negative emotions are inversely correlated with suicidal ideation (30). However, parent-child relationships and their effects on adolescent adjustment may not always be the same across cultures (24, 31). Especially in Chinese families, closeness, affect and involvement (reactive use) are not clearly defined in relation to child outcomes (32). In this sense, some studies have shown that Chinese-American adolescents whose parents are highly demanding but lowly responsive to their children adjust well, especially in terms of academic achievement (33, 34). Similar results have been found in other traditionally hierarchical societies, such as Arabia (35, 36). However, recent evidence seems to suggest that parent-child relationships based on responsiveness (communication and trust) are beneficial for adolescents even in the People's Republic of China (32). Unlike Western countries, China has a particular cultural context due to the country's special one-child policy in the 1970s and the opening up of the second child policy in recent years. Chinese adolescents have experienced the role transformation from being an only child to a non-only child. Moreover, with Chinese large population, most adolescents are under great academic pressure, so adolescent suicidal tendencies and parent-child attachment might be specific to China. As mentioned before, adolescents are at a period of relative psychosocial vulnerability for adolescents, possibly because during adolescence, peer influence on adolescents becomes increasingly important while parental influence wanes (28, 37). Although the influence of parents on adolescents at this time is not as strong as it used to be, it still plays an important role. Relevant studies show that families with conflicts are more likely to have a low parent-child attachment (38). When parental conflict arises, children perceive tension in family relationships, which in turn produces various negative emotions such as uneasiness and loss, moreover, leads to instability in parent-child attachment. A study in China showed that poor family relationships produce frequent parental conflicts, leading to feelings of insecurity and distress, which can weaken the attachment bond with their parents (11). Another study proves this view in reverse: The higher the quality of parents' marriage and mutual support, the more positive their mentality is when treating their children, and the higher their children's sense of attachment and security (39). Meanwhile, several studies have shown the complex effects of parent-child attachment on adolescents. One study found that the parent-child relationship was directly related to adolescents' academic engagement. Other results showed that the parent-child relationship indirectly predicted adolescents' academic engagement through academic motivation and academic self-efficacy (40). Another study found that parent-child attachment mediated the relationship between perceived parental conflict and social adjustment behaviors (41). Although current studies have already clarified the association between parent-child attachment and suicide intention, as well as the association between parental conflict perceptions and suicide intention, however, the mechanism of action between adolescent suicide and the variables of parent-child attachment and perceived parental conflict is unclear, and there is a lack of relevant research in the specific context of China.

Due to the contradictions in current research results and the complex mechanisms between variables, it is necessary to conduct in-depth research to further verify the relationship between variables among Chinese adolescents. We have reason to believe that a weakened parent-child attachment, resulting from perceived parental conflict, could increase an adolescent's suicide intention. Therefore, the uniqueness of this study lies in its exploration of the mediating role of parent-child attachment between parental conflict perception and suicide intention. The present study was conducted in China to explore the relationship between parent attachment and adolescents' suicide intention, and the relationship between adolescents' perceived level of parental conflict and their suicidality, as well as the relationship between parent-child attachment and perceived parental conflict, and between parental conflict and suicidal ideation. Specifically, the present study set out the following hypothesizes:

1. Is there a correlation between the suicidal intentions of adolescents in china and their parental conflict perceptions and parent-child attachment?
2. Does parent-child attachment act as a mediator between parental conflict perception and suicidal intention?

## Methods

### Study design and participants

This study was conducted in Shenyang, Liaoning Province, China. We selected two junior high schools and two senior high schools using a stratified random sampling method. The students were from grades 1 to 3 in junior high school, with an average of 4 classes in each grade. The students from grades 7 to 9 in secondary school, with an average of 6 classes in each grade. We collaborated with the schools in the recruitment of participants. School health education or health promotion staff assisted us in distributing and collecting the questionnaires, while ensuring that one investigator (a member of the research team) was assigned to each class for on-site supervision and answering questions to ensure quality control of the survey. All participants were informed about the purpose of the study and their participation was voluntary. We also obtained consent from their parents. The questionnaires were distributed to the participants during school hours with the help of the school health staff. The students were given a set amount of time to complete the questionnaires in a quiet and comfortable environment. After completion, the questionnaires were collected and checked for completeness. Overall, 1,028 participants were selected in this study. A total of 1,028 students were given questionnaires including the Suicidal Behavior Questionnaire-Revised (SBQ-R), Children's Perception of Interparental Conflict Scale (CPIC) and revised version of inventory of parent attachment (IPPA-R), excluding 132 questionnaires for missing and incomplete answers. In the end, 896 valid questionnaires were obtained. At last, the effective response rate is 87.6%. The procedure of this experiment conforms to the ethical standards of the Professional Committee of Human Experimentation of China Medical University. We informed all participants and their parents about the content and purpose of the study, and obtained their consent.

### Measures

#### Measures of suicide intention

The Suicidal Behavior Questionnaire-Revised (SBQ-R) was used to measure suicide intention in our study. SBQ-R was simplified from The Suicidal Behavior Questionnaire, which consists of four items and the scale has a total score of 3–18, the higher the score, the higher the risk of suicide. The scale has a critical score of 7. When the final score ≥ 7, the participant was at risk of suicide, contrarily, there was no risk of suicide intention (42–45). For example, the first item is “Have you ever thought about or attempted to kill yourself?” and the second item is “How often have you thought about killing yourself in the past year?” The SBQ-R has been validated in a Chinese context, showing good internal consistency with a Cronbach's alpha of 0.88 (46). In this study, we included gender and age as covariates in our model. This is because previous research has shown that these two factors may influence adolescent suicidal intention. By including them as covariates in our model, we can more accurately assess the relationships between parental conflict perception, parent-child attachment, and suicidal intention.

#### Measures of parental conflict perceptions

This study used Children's Perception of Interparental Conflict Scale (CPIC), designed by Grych et al. (47) to measure parental conflict perception. The scale has a total of 52 entries and is divided into 9 dimensions, including conflict frequency, intensity, content, resolution, perceived threats, self-blame, triangulation, response effect and attribution stability. The higher the score, the higher the child's perception of parental conflict. This scale is used to measure the frequency and intensity of parental conflict that the child perceives (47, 48). Example items were as followed: (1) “I can't make myself feel better when my mom and dad fight”; (2) “My mom and dad usually still make up after every argument”. The CPIC has been validated in a Chinese context, with a Cronbach's alpha of 0.92, indicating excellent reliability (49).

#### Measures of parent-child attachment

This study used revised version of inventory of parent attachment (IPPA-R), designed and Adapted by Armsden and Greenberg (50) to measure parent-child attachment. This scale includes two subscales father, mother attachment, and each subscale includes 25 items and 3 dimensions (trust, communication and alienation). Each item is scored on a 5-point scale from 1 to 5, from “not at all” to “completely.” In the 3 dimensions, the higher the score in the trust and communication dimensions and lower the score in the alienation dimension, the better the parent-child attachment relationship becomes. The total score of attachment is calculated as follows: Trust dimension + communication dimension - Alienation dimension (50–52). Example items were as followed: (1) “My mother respects my feelings”; (2) “I feel my mother does a good job as a mother,” (for the father's version, replace “mother” with “father”). The IPPA-R has been validated in a Chinese context, showing strong reliability with a Cronbach's alpha of 0.87 for the mother attachment subscale and 0.89 for the father attachment subscale (53).

### General information

General information includes gender, age, living place, family type, monthly household income and left-behind children. Age was divided into “10–14” group and “15–19” group. LBC are children whose one or both parents have been away from home for at least 6 months. Living area includes urban and rural area. Family type was divided into type A (immediate family or extended family) and type B (Single parent or foster care or others). Previous research has shown that family structure can have a significant impact on children's mental health. For example, children from single-parent or foster families may face different challenges compared to children from immediate or extended families, which may affect their mental health and wellbeing (54). The definition of high income was defined as earned more than 3,000 Chinese Yuan monthly (~420 USD or 390 EUR) and the definition of low income was defined as earned < 3,000 Chinese Yuan monthly (~420 USD or 390 EUR). Rural areas were defined as regions with a population density below 1,500 persons per km^2^, while urban areas were defined as those with more than 1,500 persons per km^2^.

### Statistical analysis

This study used descriptive statistics, *t-*test and chi-square test to describe and compare the main characteristics of the study subjects. Univariate and binary logistic regression models were used to estimate the association between parental conflict perceptions, parent-child attachment and suicide intention. In order to meet the needs of logistic regression analysis, the dimensions of parent-child attachment and parental perception conflicts were dichotomize based on the calculated median. Mediation analyses were carried out using the Process command for SPSS. All statistical analyses were performed using SPSS software (version 26.0) (IBM SPSS, Inc. Chicago, IL, USA), two-sided tests, and P ≤ 0.05 was considered statistically significant.

## Results

### The participant's descriptive statistics

Table 1 presents the descriptive statistics for each variable in our study, including the minimum scores, maximum scores, mean scores, and standard deviations.

**Table 1:** Presents the descriptive statistics for each variable in our study (*n* = 896).

**Variables**	**Min scores**	**Max scores**	**Mean scores**	**Standard deviation**
Age	11.000	19.000	15.384	1.408
**Parental conflict perceptions**				
Intensity	7.000	42.000	21.135	6.931
Resolution	7.000	42.000	20.772	6.742
Content	4.000	24.000	9.924	4.303
Perceived threats	6.000	36.000	19.200	6.441
Response effect	6.000	36.000	19.368	4.830
Self-blame	5.000	30.000	14.448	4.063
Triangular relationships	4.000	24.000	11.846	4.215
Attribution stability	4.000	24.000	9.295	4.456
Conflict frequency	6.000	36.000	18.177	5.767
**Parent-child attachment of mother**				
Trust	7.000	35.000	27.465	5.645
Communication	8.000	40.000	28.789	7.059
Alienation	10.000	50.000	26.370	7.472
**Parent-child attachment of mother**				
Trust	7.000	35.000	27.043	5.845
Communication	8.000	40.000	27.771	7.368
Alienation	10.000	50.000	26.580	7.585
Suicide intention	3.000	18.000	5.113	2.880

### Correlation of main variables

The correlations of the main variables are detailed in Table 2.

**Table 2:** The correlation table with the main variables (*n* = 896).

**Variables**	**1**	**2**	**3**	**4**	**5**	**6**	**7**	**8**	**9**	**10**	**11**	**12**
Intensity	1											
2. Resolution	0.688^**^	1										
3. Content	−0.512^**^	0.242^**^	1									
4. Perceived threats	0.558^**^	0.225^**^	0.498^**^	1								
5. Response effect	0.562^**^	0.448^**^	0.371^**^	0.564^**^	1							
6. Self-blame	0.345^**^	0.279^**^	0.600^**^	0.0329^**^	0.189^**^	1						
7. Triangular relationships	0.461^**^	0.188^**^	0.577^**^	0.693^**^	0.456^**^	0.365^**^	1					
8. Attribution stability	0.616^**^	0.466^**^	0.644^**^	0.502^**^	0.432^**^	0.403^**^	0.557^**^	1				
9. Conflict frequency	0.771^**^	0.662^**^	0.522^**^	0.553^**^	0.586^**^	0.338^**^	0.497^**^	0.620^**^	1			
10. Parent-child attachment of mother	−0.444^**^	−0.497^**^	−0.359^**^	−0.222^**^	−0.408^**^	−0.250^**^	−0.191^**^	−0.397^**^	−0.454^**^	1		
11. Parent-child attachment of father	−0.440^**^	−0.502^**^	−0.297^**^	−0.222^**^	−0.430^**^	−0.212^**^	−0.179^**^	−0.334^**^	−0.430^**^	−0.835^**^	1	
12. Suicide	0.246^**^	0.248^**^	0.133^**^	0.111^**^	0.217^**^	0.113^**^	0.074^**^	0.130^**^	0.201^**^	−0.349^**^	−0.367^**^	1

^*^P < 0.05 Statistically significant at confidence level of 95%. ^**^P < 0.01 Statistically significant at confidence level of 99%.

### General features of study participants

A total 92 (22.4%) of the male respondents were at high risk of suicide, while 114 (23.5%) of the female respondents were at high risk of suicide (*P* = 0.718, χ^2^ = 0.130); 55 (17.6%) respondents whose age is 10~14 were at high risk of suicide, while 151 (25.9%) of the respondents whose age is 15~19 were at high risk of suicide (*P* < 0.05, χ^2^ = 7.776). The distribution of the dependent variable varies across age groups. Although gender is not significant and age is significant in our study, epidemiologic surveys typically force both gender and age into the model as the most controlled variables. Therefore, gender and age were controlled as covariance to better screen for the effects of other variables on it. In terms of place of living place, 170 (24.7%) of the urban respondents were at high risk of suicide, while 36 (14.7%) of the rural respondents were at high risk of suicide (*P* < 0.05, χ^2^ = 4.768); 45 (32.8%) of the LBC respondents were at high risk of suicide, while 161 (21.2%) of the NLBC respondents were at high risk of suicide (*P* < 0.05, χ^2^ = 8.873); 139 (20.6%) of the respondents who lives with parents or grandparents (Type A) were at high risk of suicide, while 67 (30.5%) of the respondents who lives in a single family or other family (Type B) were at high risk of suicide (*P* < 0.05, χ^2^ = 9.147). Detailed data are presented in Table 3.

**Table 3:** The general characteristics (*n* = 896).

**Variables**	**High risk of suicide**	**Low risk of suicide**	**χ^2^**
**Gender**		
Male	92 (22.4%)	318 (77.6%)	
Female	114 (23.5%)	372 (76.5%)	0.130
**Age**		
10–14	55 (17.6%)	257 (82.4%)	
15–19	151 (25.9%)^**^	433 (74.1%)	7.776
**Living place**		
Urban	170 (24.7%)^*^	519 (75.3%)	
Rural	36 (17.4%)	171 (82.6%)	4.768
**LBC**		
Yes	45 (32.8%)^**^	92 (67.2%)	
No	161 (21.2%)	598 (78.8%)	8.873
**Family type**		
Type A	139 (20.6%)	537 (76.4%)	
Type B	67 (30.5%)^**^	153 (69.5%)	9.147
**Income**		
High	91 (23.9%)	289 (76.1%)	
Low	115 (22.3%)	401 (77.7%)	0.341

^*^P < 0.05 Statistically significant at confidence level of 95%. ^**^P < 0.01 Statistically significant at confidence level of 99%.

### Results of univariate analysis

The relationship between parental conflict perceptions, parent-child attachment and suicide intention are shown in Table 4. Among the nine dimensions of parental conflict perceptions and 3 dimensions of parent-child attachment (father/mother), respondents with high intensity (*P* < 0.05, χ^2^ = 40.343), high resolution (*P* < 0.05, χ^2^ = 30.894), high content (*P* < 0.05, χ^2^ = 10.086), high perceived threats (*P* < 0.05, χ^2^ = 9.102), high response effect (*P* < 0.05, χ^2^ = 25.820), high self-blame (*P* < 0.05, χ^2^ = 7.879), high attribution stability (*P* < 0.05, χ^2^ = 15.027), high conflict frequency (*P* < 0.05, χ^2^ = 22.693), low trust (mother: *P* < 0.05, χ^2^ = 44.200; father: *P* < 0.05, χ^2^ = 40.509), low communication (mother: *P* < 0.05, χ^2^ = 40.733; father: *P* < 0.05, χ^2^ = 38.285), high alienation (mother: *P* < 0.05, χ^2^ = 72.746; father: *P* < 0.05, χ^2^ = 68.180) are at high risk of suicide. Triangular relationships is statistically insignificant (*P* = 0.288, χ^2^ = 1.130). Detailed data are presented in Table 4.

**Table 4:** The univariate analysis (*n* = 896).

**Variables**	**High risk of suicide**	**Low risk of suicide**	**χ^2^**
**Parental conflict perceptions**			
**Intensity**			
High	143 (31.9%)^**^	305 (68.1%)	
Low	63 (14.1%)	385 (85.9%)	40.343
**Resolution**			
High	135 (31.0%)^**^	300 (69.0%)	
Low	71 (15.4%)	390 (84.6%)	30.894
**Content**			
High	123 (27.5%)^**^	325 (72.5%)	
Low	83 (18.5%)	365 (81.5%)	10.086
**Perceived threats**			
High	122 (27.2%)^**^	326 (72.8%)	
Low	84 (18.8%)	364 (81.3%)	9.102
**Response effect**			
High	135 (30.1%)^**^	313 (69.9%)	
Low	71 (15.8%)	377 (84.2%)	25.820
**Self-blame**			
High	102 (27.7%)^**^	266 (72.3%)	
Low	104 (19.7%)	424 (80.3%)	7.879
**Triangular relationships**			
High	97 (24.7%)	296 (75.3%)	
Low	109 (21.7%)	394 (78.3%)	1.130
**Attribution stability**			
High	116 (29.1%)^**^	283 (70.9%)	
Low	90 (18.1%)	407 (81.9%)	15.027
**Conflict frequency**			
High	133 (29.7%)^**^	315 (70.3%)	
Low	73 (16.3%)	375 (83.7%)	22.693
**Parent-child attachment of mother**			
High	51 (11.5%)	392 (88.5%)	
Low	155 (34.2%)^**^	298 (65.8%)	
**Mother trust**			
High	55 (13.1%)	366 (86.9%)	
Low	151 (31.8%)^**^	324 (68.2%)	44.200
**Mother communication**			
High	56 (13.4%)	362 (86.6%)	
Low	150 (31.4%)^**^	328 (68.6%)	40.733
**Mother alienation**			
High	153 (35.4%)^**^	279 (64.6%)	
Low	53 (11.4%)	411 (88.6%)	72.746
**Parent-child attachment of father**			
High	49 (10.9%)	399 (89.1%)	73.526
Low	157 (35.0%)^**^	291 (65.0%)	
**Father trust**			
High	62 (14.0%)	382 (86.0%)	
Low	144 (31.9%)^**^	308 (68.1%)	40.509
**Father communication**			
High	57 (13.7%)	360 (86.3%)	
Low	149 (31.1%)^**^	330 (68.9%)	38.285
**Father alienation**			
High	155 (34.6%)^**^	293 (65.4%)	
Low	51 (11.4%)	397 (88.6%)	68.180

^*^P < 0.05 Statistically significant at confidence level of 95%. ^**^P < 0.01 Statistically significant at confidence level of 99%.

### The logistic regression analysis

The significant variables in Tables 3, 4 were incorporated into the logistic regression model. Parent-child attachment of mother and father used the total score. All analysis of variables that do not have significance is not listed. In this study, we included gender and age as covariates in our model. Table 5 shows that the negative factors for suicide were age between 15 and 19 years (OR = 1.595, *P* < 0.05) and high sense of parental conflict (OR = 1.688, *P* < 0.05); the positive factors for suicide included residence in a rural area (OR = 0.474, *P* < 0.01), high degree of mother's parent-child attachment (OR = 0.568, *P* < 0.05) and high degree of father's parent-child attachment was high (OR = 0.436, *P* < 0.01).

**Table 5:** The logistic regression analysis for exploring factors of suicide risk (*n* = 896).

**Variables**	**Suicide risk**		
	**OR**	**95% CI**	
Age (15–19 vs. 10–14)	1.595^*^	1.094	2.327
Living place (rural vs. urban)	0.474^**^	0.304	0.740
Strength of parental conflict perceptions (high vs. low)	1.688^*^	1.036	2.686
Parent-child attachment of mother (high vs. low)	0.568 ^*^	0.337	0.959
Parent-child attachment of father (high vs. low)	0.436^**^	0.259	0.733

^*^P < 0.05 Statistically significant at confidence level of 95%. ^**^P < 0.01 Statistically significant at confidence level of 99%.

### Mediation analysis

#### The results of mediating analysis of parent-child attachment between resolved situations and suicide intention

Table 6 shows the results of mediating analysis of parent-child attachment between Resolved situations and suicide intention. First, the total effect between Resolved situations and suicide intention is significantly relevant (LLCI~ULCI:0.0091~0.00971), then, the direct effect between Resolved situations and suicide intention has no significant correlation (LLCI~ULCI:−0.0384~0.0495). Last, mother attachment and father attachment were included in our study model as mediating variables, respectively and, indirect effects analysis is also performed. The result shows that the Indirect effect of mother attachment is significantly relevant (LLCI~ULCI: 0.0025~0.0036) and the Indirect effect of father attachment is also significantly relevant (LLCI~ULCI: 0.0139~0.0486). Thus, mother attachment, father attachment act as a complete intermediary in the relationship between Resolved situations and suicide intention. The significance of the indirect effect was tested using bootstrapping.

**Table 6:** Mediating analysis of parent-child attachment between resolved situations and suicide intention (*n* = 896).

	**Effect**	**SE**	**t**	***P***	**LLCI**	**ULCI**
Total effect	0.0531	0.0224	2.3690	0.0181	0.0091	0.0971
Direct effect	0.0056	0.0224	0.2489	0.8035	−0.0384	0.0495
Indirect effect of mother attachment	0.0183	0.0081			0.0025	0.0346
Indirect effect of father attachment	0.0292	0.0088			0.0139	0.0486

All dimensional analyses that do not have significant correlations are not listed.

#### The results of mediating analysis of parent-child attachment between response effect and suicide intention

Table 7 shows the results of Mediating analysis of parent-child attachment between response effect and suicide intention. The total effect between response effect and suicide intention (LLCI~ULCI: 0.0274~0.1282); The direct effect between response effect and suicide intention has no significant correlation (LLCI~ULCI: −0.0163~0.0834). Last, mother attachment and father attachment were included in our study model as mediating variables, respectively and, indirect effects analysis is also performed. The result shows that the Indirect effect of mother attachment is significantly relevant (LLCI~ULCI: 0.0014~0.0313) and the Indirect effect of father attachment is also significantly relevant (LLCI~ULCI: 0.0130~0.0491). Thus, mother attachment, father attachment act as a complete intermediary in the relationship between response effect and suicide intention.

**Table 7:** Mediating analysis of parent-child attachment between response effect and suicide intention (*n* = 896).

	**Effect**	**SE**	**t**	***P***	**LLCI**	**ULCI**
Total effect	0.0778	0.0257	3.0293	0.0025	0.0274	0.1282
Direct effect	0.0335	0.0254	1.3206	0.1870	−0.0163	0.0834
Indirect effect of mother attachment	0.0153	0.0076			0.0014	0.0313
Indirect effect of father attachment	0.0289	0.0093			0.0130	0.0491

All dimensional analyses that do not have significant correlations are not listed.

In Tables 6, 7, we present the results of the mediation analysis of parent-child attachment between resolved situations and suicide intention. The total effect represents the overall association between resolved situations and suicide intention, the direct effect represents the direct impact of resolved situations on suicide intention, and the indirect effect represents the mediating role of parent-child attachment between resolved situations and suicide intention. LLCI and ULCI represent the 95% confidence interval of the effect, and if the confidence interval does not contain 0, it indicates that the effect is significant.

According to our analysis results, there is a significant total effect between resolved situations and suicide intention, but the direct effect is not significant. However, when mother attachment and father attachment are used as mediating variables, their indirect effects are both significant. This indicates that parent-child attachment plays a complete mediating role between resolved situations and suicide intention.

These results have important implications for both theory and practice. They suggest that parent-child attachment plays an important role in resolving conflicts and preventing adolescent suicide intention. Therefore, in practice, we can help adolescents better resolve conflicts and reduce suicide intention by promoting parent-child attachment. In future research, we can further explore effective ways to promote parent-child attachment, as well as other possible mediating variables.

## Discussion

A lot of studies have shown that suicide is serious all around the word (54–56). However, most studies have explored risk factors for suicide only in terms of the suicide population itself and rarely conducted from their family factors. This study used the univariate analysis and the binary logistics regression to analyze various risk factors for suicide, including their own factors and family environmental factors Furthermore, Mediation analysis was used to explore the mediating effects of father and maternal mother attachment as mediating variables between the 9 dimensions of parental conflict and suicide intention.

Our study shows that parent-child attachment (father attachment and mother attachment) plays a completely mediating role in the connection between two dimensions of parental conflict perception (Resolved situations, response effect) and suicide intention. A study showed that there is a negative correlation between parental marital conflict and attachment between parents and children (57). At the same time, there are some studies which have proven that One's attachment to his/her father determines how likely one is to consider suicide (58). This result can be interpreted as the better the resolution and response effect of parental conflict, the better the parent-child attachment becomes, the lower the risk of suicide keeps. The probable reason for this result may be due to the fact that a good resolution and a good response effect in a conflict lead to a high degree of parent-child attachment, a high degree of parent-child attachment can significantly reduce the risk of suicide in adolescents (59). Our study findings align with research conducted on Western families, particularly from the United States (24–26) and Europe (18, 28), indicating the significance of a parent-child relationship built on responsiveness. This relationship fosters enhanced communication, trust, and reduced feelings of alienation, serving as a protective buffer against suicidal ideation. These cross-cultural similarities underscore the universal importance of parental responsiveness in mitigating adolescent suicide risk. Therefore, positive response and reasonable resolution of family conflicts can effectively improve the degree of parent-child attachment, thereby reducing the risk of suicide in adolescents. This finding provides us with a deeper understanding about adolescent suicide and helps us better develop suicide prevention strategies.

The relationship between the age of adolescents and suicide risk remains unclear in the research literature. Some studies have found a higher risk of suicide among younger adolescents, while others have reported a higher risk among older adolescents. Our research indicates that respondents aged 15–19 have a higher risk of suicide. This may be due to the fact that older adolescents, compared to their younger counterparts, are closer to puberty, are more emotionally sensitive, and are more likely to have various psychological issues (60). This study also found that Adolescents living in rural areas are at significantly lower risk of suicide than their urban peers. The reason for this may be that urban children face far greater academic pressure than rural children and the pace of life is much faster than in the countryside (61). In addition, the interpersonal relationship in the countryside is better than in the city, the resulting sense of apathy can also lead to psychological problems, and even affect an individual's behavior and eventually lead to suicide (62). Also, this study found that the respondents with strong parental conflict perceptions suicidal intention is higher than those who has weak parental conflict perceptions. This conclusion is in line with some relevant foreign studies (63). The reason may be that adolescents who perceived strong parental conflict perceptions can lead to various emotional problems such as depression, anxiety along with problem behaviors such binge drinking, self-harm and Drug, eventually leads to suicide (64–66).

Moreover, our results challenge previous classical research in China which suggested that a parent-child relationship characterized by lower responsiveness may benefit Chinese adolescents (33, 34). Instead, our findings align more closely with recent evidence suggesting that, within Chinese society, parental responsiveness plays a vital role in safeguarding adolescents against various challenges (32). The reason for these interesting results may be due to the special one-child policy enacted in China from 1979, where parents paid excessive attention to and doted on their children, which in turn led to the children's psychology of resisting rebellion and wanting to get rid of parental constraints and control, so instead, to a certain extent less attached parent-child relationships would promote children's growth (67), but with the changes in China's fertility policy, the proportion of adolescents who are not one-child children is now considerably higher than before, and there have been changes in the structure of the family, coupled with the fierce competition in society and the parents' busy schedule, which may result in the negligence of their children (68). Today's children need more close parent-child relationships so that close parent-child relationships would promote children's growth.

Through this study, we also found that both father attachment and mother attachment are the protective factors for suicide. Teenagers with high parent-child attachment scores have low risk of suicide, which is consistent with the conclusions of the relevant studies (69). The reason is that high-quality parent-child attachment will lead to high social adaptability and cultivate positive psychological qualities, in turn, it reduces various negative emotions and bad behaviors including suicide (59). LBC is also a high risk of suicide, the reason for this may be that LBC lacks parental love from an early age, which can lead to multiple psychological problems, which leads to a higher risk of suicide (70–73). Therefore, in order to reduce suicide among adolescents, effective measures such as improve the psychological quality and anti-stress of adolescents, reduce academic pressure and enrich extracurricular activities, reduce family conflicts and improve parent-child relationships should be taken.

Besides, each of the demographic variables in our study could have the following potential effects on psychometrics: Age affects psychometrics as it relates to different stages of cognitive and emotional development. Younger adolescents may interpret and react to situations differently compared to older adolescents (74). Due to societal gender norms and expectations, males and females may have different experiences and perspectives (75), which may affect their responses on psychometric measures. However, there are also studies showing that psychometric measures do not differ between men and women, and this is consistent with our results (76). Further studies are needed to further confirm this relationship. Where one lives can affect psychometric measures because urban and rural environments can present different experiences and challenges. For example, adolescents living in urban areas may face more academic and social pressures, whereas adolescents living in rural areas may face more economic pressures and lack of resources (77). Family type can affect psychometric outcomes because different family structures provide different levels of support and stability. For example, adolescents from single-parent or foster families may face different challenges compared to those from immediate or extended families (54), which may affect their responses on psychometric measures.

## Limitations

This study has several limitations. First, the study used a cross-sectional survey design. There is no definitive conclusion in causation and it is impossible to determine whether the effects of each factor on adolescents over time are still present, so further longitudinal studies should be conducted. Second, the study used questionnaires to survey adolescents, and respondents' responses may be biased from reality. Gathering information from multiple sources, including adolescents, teachers, and parents, will contribute to future research. Despite these limitations, this study was conducted in Shenyang, one of the largest provincial capitals of China, and the sample is well represented. And in this study, the parent-child attachment was innovatively used as a mediating variable for analysis, the results of the study provide instructive recommendations for reducing adolescent suicidal intentions.

## Implications for research and practice

Adolescents Suicide is one of the gravest global public health events of recent decades which has a significant impact on the economic development of the country and the future of society, especially in developing countries. Our study examined the relationship among parent-child attachments, parental conflict perception and suicide, and parent-child attachments in mediation between parental conflict perception and suicide, aimed at arousing the awareness of the country, society, schools and families, so as to take effective measures to reduce the suicide rate among adolescents. As well as providing theoretically relevant data, our findings have practical implications as well. Firstly, our research was from the point of view of the family environment, has revealed the psychological damage of conflicts between parents to adolescents, provided a rationale for family-school cooperation for the prevention of adolescent suicide. Secondly, our study was conducted by primary and secondary school students in China, grouping special groups of left-behind children, providing opinions and suggestions for mental health intervention and suicide prevention for left-behind children in urban and rural areas. Thirdly, the results enriched the family perspective of adolescent suicide-related research, and helped to understand the relationship between perceptual parental conflict and adolescent suicide from the perspective of parent-child attachment.

## Conclusion

Our research results have shown that teenagers that is LBC, aging 15–19, living in urban and having strong parental conflict perceptions and low Parent-child attachment have a high risk of suicide in the population. Parent-child attachment plays completely mediating role in the connection between 2 dimensions of parental conflict perception (Resolved situations and response effect) and suicide. These high-risk factors for suicide should be targeted and active and effective measures such as improve the psychological quality and anti-stress of adolescents, reduce academic pressure and enrich extracurricular activities, reduce family conflicts and improve parent-child relationships should be taken to escort the future of adolescents.

## Data availability statement

The raw data supporting the conclusions of this article will be made available by the authors, without undue reservation.

## Ethics statement

The studies involving humans were approved by Human Experimentation of China Medical University. The studies were conducted in accordance with the local legislation and institutional requirements. Written informed consent for participation in this study was provided by the participants' legal guardians/next of kin.

## Author contributions

JW: Funding acquisition, Writing—original draft, Writing—review & editing. KC: Formal analysis, Writing—review & editing. XH: Software, Writing—review & editing. ZJ: Data curation, Supervision, Writing—review & editing. JH: Validation, Writing—review & editing. BH: Data curation, Methodology, Writing—review & editing. LF: Methodology, Writing—review & editing. NM: Software, Writing—review & editing. CY: Data curation, Supervision, Writing—review & editing. PY: Investigation, Supervision, Writing—review & editing. ZL: Investigation, Supervision, Writing—review & editing.
